# Response of *Moringa oleifera* Seeds and Fixed Oil Production to Vermicompost and NPK Fertilizers under Calcareous Soil Conditions

**DOI:** 10.3390/plants10101998

**Published:** 2021-09-24

**Authors:** Amira K. G. Atteya, Aishah N. Albalawi, Rasha S. El-Serafy, Khalil N. Albalawi, Hala M. Bayomy, Esmail A. E. Genaidy

**Affiliations:** 1 Horticulture Department, Faculty of Agriculture, Damanhour University, Damanhour 22516, Egypt; 2 Department of Analytical Chemistry, Tabuk University, University College of Haql, Tabuk 71491, Saudi Arabia; an.albalawi@ut.edu.sa; 3 Horticulture Department, Faculty of Agriculture, Tanta University, Tanta 31527, Egypt; rasha.elserafi@agr.tanta.edu.eg; 4 Prince Sultan Military College for Health Sciences, Dhahran 34313, Saudi Arabia; Knawaf@psmchs.edu.sa; 5 Department of Nutrition and Food Science, Tabuk University, Tabuk 71491, Saudi Arabia; hala.biomy@agr.dmu.edu.eg; 6 Department of Food Science and Technology, Damanhour University, Damanhour 22516, Egypt; 7 Pomology Department, National Research Centre, Giza 12622, Egypt; esmail_nrc@yahoo.com

**Keywords:** *Moringa oleifera* production, pods, seeds, and fixed oil yield, fatty acids, calcareous soil, vermicompost, nanofertilizers

## Abstract

A shortages of soil nutrients resources and a lack of accessibility to them especially in calcareous soil are considered some of the main factors that limit plant production. In particular, the cultivation of the *Moringa oleifera* trees in this type of soil is of special interest given the increasing demand for every part of this tree. Several studies have focused on the production of its leaves as an herbaceous plant and not as a tree, but there has not been extensive research on its pods, seeds, and fixed oil production. In this sense, in this study, we provide an assessment of the use of fertilizers, vermicompost and NPK (as traditional minerals and as nanoparticles), in order to improve pods, seeds, and fixed oil contents, as indicators of the quality of the production of the *Moringa oleifera* trees in calcareous soil conditions. In this experiment, it was observed that all parameters and the yield of pods, seeds, and fixed oil of the *Moringa oleifera* tree were significantly improved by increasing the level of vermicompost and using NPK fertilization and combination treatments in both seasons of the study. The combination treatments of 10 and 20 ton ha^−1^ vermicompost plus NPK control produced the highest percentage of oleic acid with insignificant differences between them.

## 1. Introduction

*Moringa oleifera* is a small or medium-sized tree, about 10 m high. It is commonly known as a horseradish or drumstick tree and all its parts are useful. It belongs to the Moringaceae family, which consists of the single genus *Moringa*, comprised of 10–14 species. The best-known species is *Moringa oleifera*, which is indigenous to northwest India and widely cultivated in the Philippines, Thailand, Malaysia, Pakistan, and other tropical and subtropical areas in Central Asia, America, and Africa [1,2], and is now cultivated in small areas and private gardens in Egypt. The *Moringa oleifera* tree is nutritional and rich in vitamins and minerals [3]. In addition, recently, its seeds have gained a attention as a source of plant oil rich in oleic fatty acid. This oil is semi-solid and yellowish-brown with a bitter almond-like odor [4]. High-oleic oils are gaining importance, especially as a replacement for polyunsaturated vegetable oils, and have been reported to exhibit good oxidative stability during frying [5]. This oil is suitable for both human consumption and commercial purposes, and therefore the *Moringa oleifera* tree is very useful in animal feed, alley cropping, household cleaning agents, biogas, medicine, green manure, ornamental plants, gum and water purification [6]. Moreover, many diseases, such as high blood pressure, lung diseases, and skin infections can be treated with different parts of the *Moringa oleifera* tree such as the leaves, inflorescences, seeds, and roots [7].

Soil is the main support for plants in agriculture, due to its contribution of nutrients through the soil solution and its function of providing physical support for plants. It is preferable to culture the *Moringa oleifera* tree in slightly acidic to neutral well-drained loam to clay loam soils [6]. Furthermore, suitable fertilization can aids the rapid growth of *Moringa oleifera* tree and enhance its ability to give a healthy plant [8] that will produces an economic yield of pods, seeds, and fixed oil. 

However, agricultural production in calcareous soils faces many difficulties due to a high level of calcium carbonate; high infiltration rate; low water holding capacity; low organic matter and clay content, poor structure, low cation-exchange capacity, surface crusting and cracking; loss of nutrients via leaching or deep percolation; high pH with a nutritional imbalance between elements such as potassium, magnesium, and calcium; and low availability of nutrients, in particularly phosphorous, iron, and micronutrients. These difficulties can result in failure to obtain the desired plant growth and yield [9,10,11,12]. Potassium has a clear role in most biochemical and physiological processes related to plant growth, productivity, and resistance to drought and disease as it is able to regulates osmotic conditions, enhances photosynthesis, and promotes carbohydrate metabolism. Therefore, plants require quite large amounts of K to produce economic crops and to promote the adaptive plant responses of plants to the environment [13]. 

Nitrogen is the main element to provide plants with the required amino acid and protein in plant. Under the alkaline conditions of calcareous soil, the rate of N transformation increases and the efficiency of N use by plants can be influenced. Therefore, minimizing ammonia volatilization and leaching of N presents the proper N management for fertilization in calcareous soil [13,14]. Phosphorus is an essential macronutrient that can improve metabolism, plant growth, root growth, flowering, maturity of seed and fruit yield, and the degree of improvement increases when phosphorus is applied in combination with nitrogen [15,16]; In addition, with alkaline pH conditions, the availability of both native and added phosphorus decreases as its anions form limited solubility compounds of P, Mg, and Ca [17]. Under such conditions, finding an approach to improve soil chemical and physical properties and a successful method to give growing plants the fertilizers they need without losses is vital for the rapid vegetative growth of the *Moringa oleifera* trees. Under these conditions, foliar fertilization is important as a part of fertilization programs for the *Moringa oleifera* trees, especially using nanofertilizers in a nanoscale range of 1–100 nm that allows them to penetrate the plant tissues. In this field, foliar application of nano-NPK fertilizers reduces soil pollution and obviates the interaction of nutrients with water, microorganisms, and calcareous soil, and therefore reduces the amount of nutrients used to below recommended levels [18]. In their studies by Gohari and NoorhosseiniNiyaki [19], Sheykhbaglou et al. [20], Bozorgi [21], and Hagagg et al. [22], the authors emphasized the benefits of nano-fertilizers. 

Vermicompost is one of the most favorable non-chemical plant nutritional sources. It can be produced through vermicomposting of organic material by earthworms, which can consume a wide range of organic residues. It has a positive influence on the physical and chemical structure of soil as well as plant growth [23] by improving the stability of soil aggregates, as well as water retention, bulk density and porosity, and soil thermal dynamics. In addition, it stimulates and increases the absorption of nutrients by plants and favors a biological control of bacterial and fungal diseases in plants [24]. Vermicompost can improve plant growth in calcareous soil in four ways:

(i) Vermicompost increases the availability of plant nutrients in soil by adding N, P, K, as well as other micro- and macro-nutrients during the process of mineralization of organic matter. Vermicompost contains a larger group of soil-friendly fungi, bacteria and actinomycetes [25,26], such as nitrogen-fixing anaerobes [27], microbes that are responsible for nitrogen mineralization and conversion of ammonium nitrogen into available plant forms [28], as well as phosphate-dissolving bacteria such as *Pseudomonas striata* [29], as plants can uptake phosphate only in a soluble ionic form [30]. Herencia et al. [31] reported that the use of organic fertilizer enriched s soil with N, available P and K as well as organic matter. Additionally, decreased availability of soil Mn, Fe, Cu, B and Zn has been associated with calcareous soils [32], which resulted from interactions with soil carbonates and pH effects [33].

(ii) Vermicompost stimulates growth-promoting hormones such as auxins, gibberellins, and cytokinins that are produced by microorganisms in vermicompost [34,35], which improve plant growth and increases plant tolerance to biotic and abiotic stresses [36]. The results of a structural analysis by Canellas et al. [37] revealed the presence of exchangeable auxin groups in the macrostructure of the humic acid fraction of vermicompost, which were found to aid in the root growth and development of *Zea mays*.

(iii) Vermicompost provides biological control of plant and soil pathogens. 

(iv) Soil health is improved by the organic matter content in vermicompost. Generally, the addition of vermicompost as an organic source to calcareous soil increases the nutrient cycling; retains soil water, soil structure, and biological processes [38,39,40,41]; and improves rooting by activating the plasma membrane H+−ATPase, by increasing acidification of the roots’ external medium [42,43]. Moreover, the low speed of nutrients degradation and release from vermicompost fertilizer is suitable for perennial plants and trees grown in calcareous soil, such as the *Moringa oleifera* trees in this study. Furthermore, organic fertilizers are suitable for achieving the objectives of sustainable agriculture as noted by many researchers, such as Arancon et al. [44], Joshi and Vig [45], Salehi et al. [46], Madahi [47], and Oftadeh [48] who studied the effect of vermicompost on plant growth. They observed that increasing the amount of vermicompost used significantly increased seeds germination, the number, length, and fresh weight of leaves; chlorophyll a, b, and total chlorophyll; growth and flowering; fruit and seed yield; and the yield of some secondary products of medicinal and aromatic plants. Additionally, Vengadaramana and Jashothan [49] reported that the addition of organic fertilizer improved soil properties.

Despite the enormous potential of the *Moringa oleifera* trees, several studies, such as those by Sánchez et al. [50], Isaiah [51], and Dania et al. [52], have treated the *Moringa oleifera tree* as an herbaceous plant, not a tree, and therefore they have focused on its leaf production under normal conditions. However, there has not been exhaustive research on the mature tree and its pods, seeds, and fixed oil production. Consequently, this study was carried out to assess the response of the *Moringa oleifera* tree to different fertilization rates using vermicompost and NPK (mineral and nanoparticles), in order to determine, under calcareous soil conditions, the rate that corresponds to optimum pod, seed, and fixed oil yield, as well as the percentage of oleic fatty acid.

## 2. Results

### 2.1. Parameters of Mature Pods

**Figure 1:**
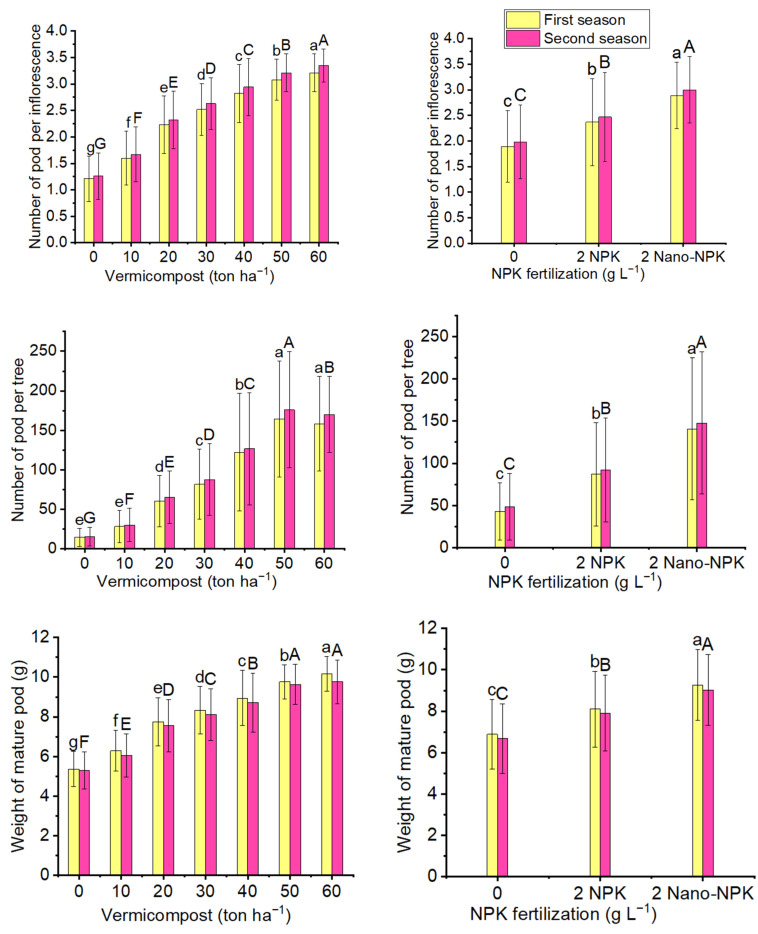
The mean values of the number of pods per inflorescence, the number of pods per tree, and the weight of mature pods (g) of the *Moringa oleifera* tree as affected by organic and mineral fertilization treatments in both seasons of the study. Data are mean values ± SE (*n* = 3). Bars with same lowercase are not significant at the *p* ≤ 0.05 level.

The parameters of mature pods of the *Moringa oleifera* trees were significantly affected by different treatments of vermicompost, NPK, and combination treatments (Figure 1 and Table 1 and Table 2). The results indicate that the 60 ton ha^−1^ vermicompost treatment produced the highest average number of pods per inflorescence (3.21 and 3.35 in the first and second seasons, respectively); the weight of mature pods (10.17 and 9.76 g), and the maximum mean values of number of pods per tree (164.6 and 176.1) in the first and second seasons respectively, were reduced by adding 50 ton ha^−1^ vermicompost across all NPK levels. In addition, when nano-NPK was used the maximum mean values of the number of pods per inflorescence (2.89 and 3.00), the number of pods per tree (140.6 and 147.6), and the weight of mature pods (9.27 and 9.03 g) were obtained across all vermicompost levels, in the first and second seasons, respectively. The interaction between vermicompost and NPK treatments was highly significant. Moreover, the 60 ton ha^−1^ vermicompost plus 2 gL^−1^ nano-NPK treatment resulted in the maximum highly significant mean values of the number of pods per inflorescence (3.53 and 3.69) and the weight of mature pods (11.22 and 10.93 g), in the first and second seasons, respectively; the maximum mean values of the number of pods per tree (243.8 and 263.5, in the first and second seasons, respectively) were produced with the 50 ton ha^−1^ vermicompost plus 2 gL^−1^ nano-NPK combination treatment (Table 1 and Table 2).

### 2.2. Yield of Mature Pods

**Figure 2:**
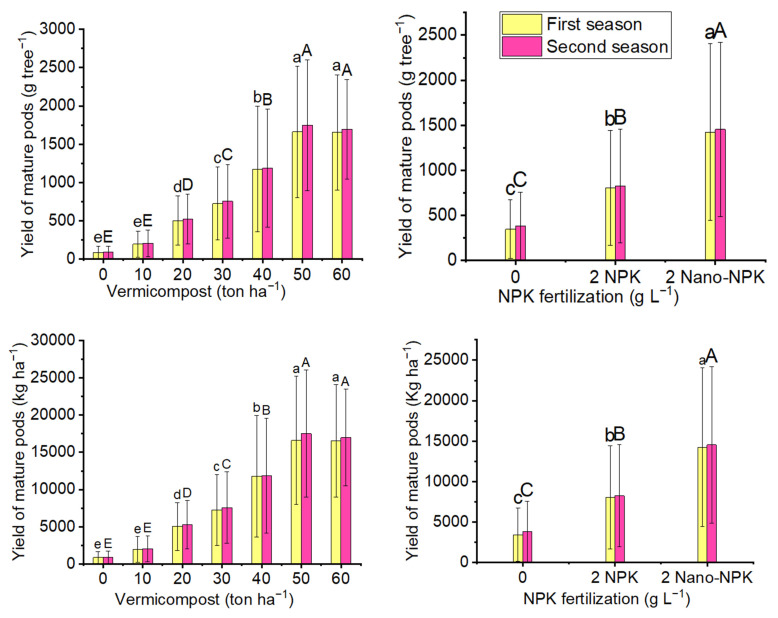
The mean value of yield of mature pods (g tree^−1^) and yield of mature pods (kg ha^−1^) of *Moringa oleifera* tree as affected by organic and mineral fertilization treatments in both seasons of the study. Data are mean values ± SE (*n* = 3). Bars with same lowercase letters are not significant at the *p* ≤ 0.05 level.

The results of the highly significant effects of different treatment levels of vermicompost, NPK, and their combinations on the yield of mature pods of the *Moringa oleifera* trees are shown in Figure 2 and Table 3. On the one hand, the 50 ton ha^−1^ vermicompost treatment across all NPK levels produced the highest average yields of mature pods per tree (1661 and 1749 g tree^−1^) and per hectare (16,605 and 17,493 kg ha^−1^) in the first and second seasons, respectively. On the other hand, by using the 50 or 60 ton ha^−1^ vermicompost treatments, insignificant differences were found in the mean values of mature pods per tree and per hectare, in both seasons. Regarding NPK fertilizer, the 2 gL^−1^ Nano-NPK treatment resulted in the maximum mean values of yield of mature pods per tree (1425 and 1454 g tree^−1^) and per hectare (14,252 and 14,537 kg ha^−1^) in the first and second seasons, respectively, across all vermicompost levels. The interaction between vermicompost and NPK treatments was high significant. Moreover, the 50 ton ha^−1^ vermicompost plus 2 gL^−1^ nano-NPK treatment produced the maximum mean values of yields of mature pods per tree (2616 and 2750 g tree^−1^) and per hectare (26,158 and 27,496 kg ha^−1^) in the first and second seasons, respectively.

### 2.3. Parameters of Mature Seeds

**Figure 3:**
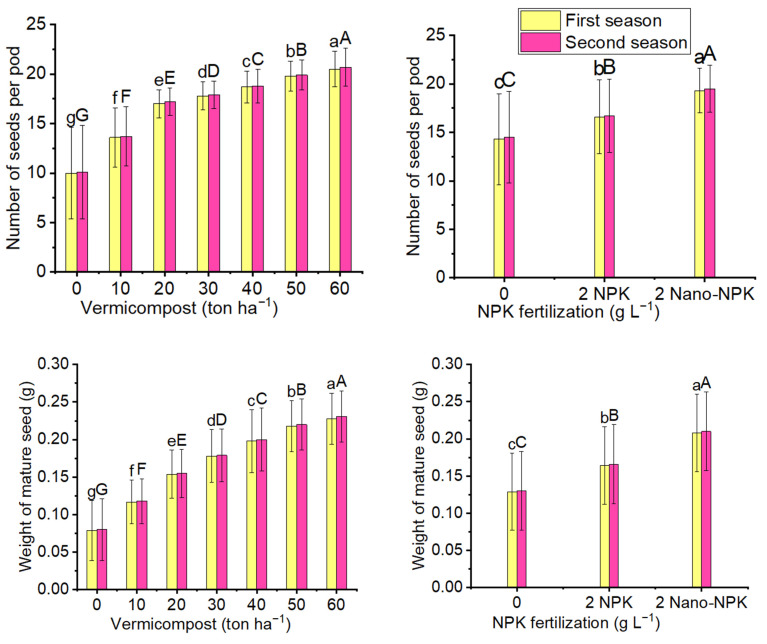
The mean values of the number of seeds per pod and weight of mature seed weight (g) of the *Moringa oleifera* tree as affected by organic and mineral fertilization treatments in both seasons of the study. Data are mean values ± SE (*n* = 3). Bars with same lowercase letters are not significant at the *p* ≤ 0.05 level.

The mean values with the highest significance for the number of seeds per pod (20.5 and 20.7) and mature seeds (0.228 and 0.231 g) in the first and second seasons, respectively, were produced by the 60 ton ha^−1^ vermicompost treatment as compared with the other vermicompost treatments, including the control. The same situation was observed for the 2 gL^−1^ nano-NPK treatment which recorded the maximum mean values of the number of seeds per pod (19.3 and 19.5) and weight of mature seed weight (0.208 and 0.210 g) as compared with the treatments of 2 gL^−1^ and the NPK control in both seasons. Regarding the combination treatments, the 60 ton ha^−1^ vermicompost plus 2 gL^−1^ Nano-NPK treatment produced the significant maximum mean values of the number of seeds per pod (22.8 and 23.0) and weight of mature seed weight (0.267 and 0.270 g) in the first and second seasons, respectively (Figure 3 and Table 4).

### 2.4. Yield of Mature Seeds

**Figure 4:**
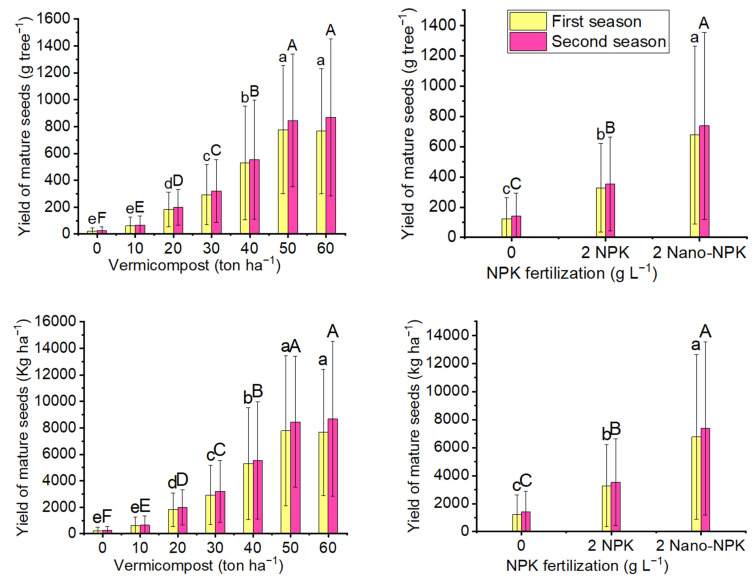
The mean values of mature seeds yield (g tree^−1^) and yield of mature seeds yield (kg ha^−1^) of the *Moringa oleifera* tree as affected by organic and mineral fertilization treatments in both seasons of the study. Data are mean values ± SE (*n* = 3). Bars with same lowercase letters are not significant at the *p* ≤ 0.05 level.

As compared with the control treatment, different levels of vermicompost, NPK, and their combinations showed significant improvements in the yield of mature *Moringa oleifera* seeds per tree and per hectare (Figure 4 and Table 5). In the first season, the mean values of yield of mature seeds per tree (777 g tree^−1^) and per hectare (7777 kg ha^−1^) was maximum with 50 ton ha^−1^ vermicompost treatment. In the second season, the 60 ton ha^−1^ vermicompost treatment produced the highest mean yield of mature seeds per tree (868 g tree^−1^) and per hectare (8678 kg ha^−1^) as compared with the other vermicompost treatments including the control. For both seasons, the differences between the mean yield of mature seeds per tree and per hectare with the 50 and 60 ton ha^−1^ vermicompost treatments were insignificant. Regarding the use of NPK, it was observed that spraying 2 gL^−1^ nano-NPK gave the maximum mean values of yields of mature seeds per tree (676 and 737 g tree^−1^) and per hectare (6759 and 7370 kg ha^−1^), in the first and second seasons, respectively, as compared with the 2 gL^−1^ NPK treatment and NPK control. Regarding the combination treatments, the 50 ton ha^−1^ vermicompost plus 2 gL^−1^ nano-NPK treatment gave the maximum yields of mature seeds yield per tree (1350 and 1482 g tree^−1^) and per hectare (13,501 and 14,821 kg ha^−1^) in the first and second seasons of the study, respectively.

### 2.5. Fixed Oil Percentage

**Figure 5:**
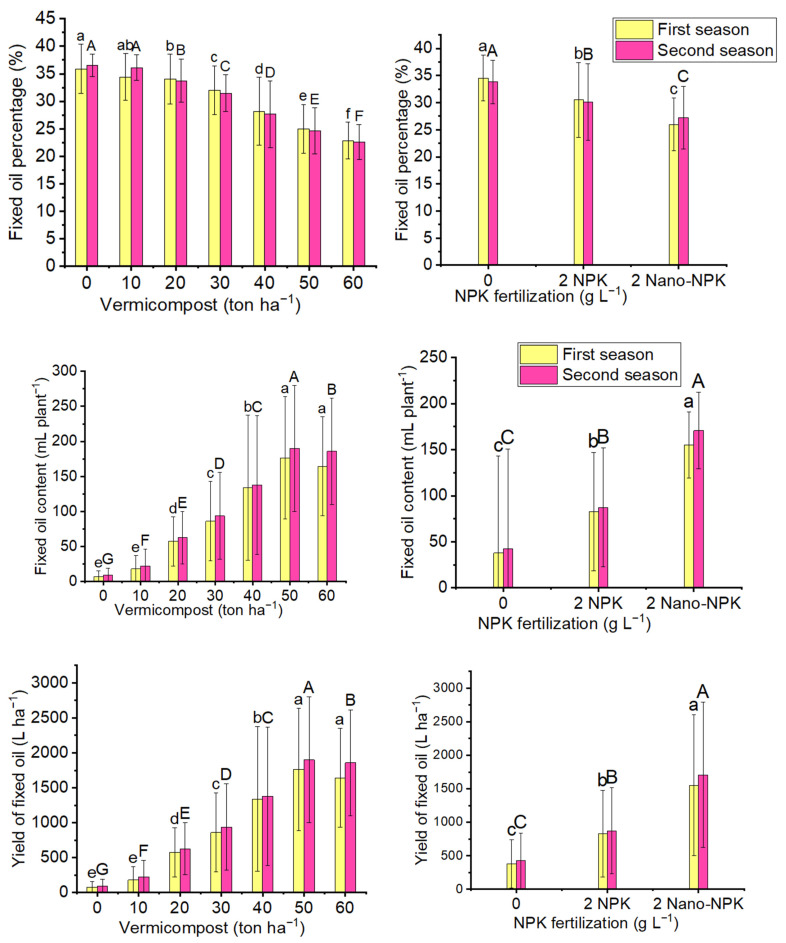
The mean values of fixed oil percentage (%), fixed oil content (mL plant^−1^), and yield of fixed oil (l ha^−1^) of the *Moringa oleifera* tree as affected by organic and mineral fertilization treatments in both seasons of the study. Data are mean values ± SE (*n* = 3). Bars with same lowercase letters are not significant at the *p* ≤ 0.05 level.

The fixed oil percentage of *Moringa oleifera* seeds was significantly affected by most levels of applied vermicompost, NPK, and their combinations treatments in both seasons (Figure 5 and Table 6). Contrary to the previously mentioned parameters, the fixed oil percentage of *Moringa oleifera* seeds in this study was decreased by increasing the vermicompost level. The vermicompost control had the maximum fixed oil percentage (35.89 and 36.50%). Insignificant differences were detected between the control and the 10 ton ha^−1^ vermicompost treatment, in both seasons. The same situation was found for the effect of NPK on fixed oil percentage as the NPK control gave the maximum highly significant percentage of fixed oil (34.53 and 33.83%) as compared with 2 gL^−1^ NPK or nano-NPK in both seasons. For the combination treatments, the second treatment of vermicompost control plus 2 gL^−1^ NPK gave the maximum Fixed oil percentage of *Moringa oleifera* seeds (38.69 and 37.48%) with insignificant differences between this result and that of vermicompost control plus NPK control in the first and second seasons, respectively.

### 2.6. Fixed Oil Yield

The variability of seeds fixed oil yield per tree and per hectare of field grown the *Moringa oleifera* tree in response to treatment with vermicompost and NPK and their combination is presented in Figure 5 and Table 6 and Table 7. The application of 50 ton ha^−1^ vermicompost recorded the maximum mean values of fixed oil yield per tree (176.4 and 189.9 mL plant^−1^) and per hectare (1764 and 1899 L ha^−1^) in the first and second seasons, respectively, as compared with the other vermicompost levels and control. In the first season, there were insignificant differences between the applications of 50 and 60 ton ha^−1^ vermicompost in terms of fixed oil yield per tree and per hectare in the first season. NPK fertilization was able to increase fixed oil yield per tree and per hectare successfully in the first and second seasons as compared with the control treatment. Moreover, the foliar application of Nano-NPK produced the maximum mean values of fixed oil yield per tree (155.1 and 170.7 mL plant^−1^) and per hectare (1551 and 1707 L ha^−1^) in the first and second seasons, respectively. For the combination treatments, the application of 50 ton ha^−1^ vermicompost plus 2 gL^−1^ nano-NPK by spraying gave the maximum mean values of fixed oil yield per tree (270.5 and 299.1 mL plant^−1^) and per hectare (2705 and 2991 L ha^−1^) in the first and second seasons, respectively. Insignificant differences were detected among T15, T18, and T21 treatments in fixed oil yield per tree and per hectare in the first season of the study.

### 2.7. Fixed Oil Analysis

#### 2.7.1. Saturated Fatty Acids

**Figure 6:**
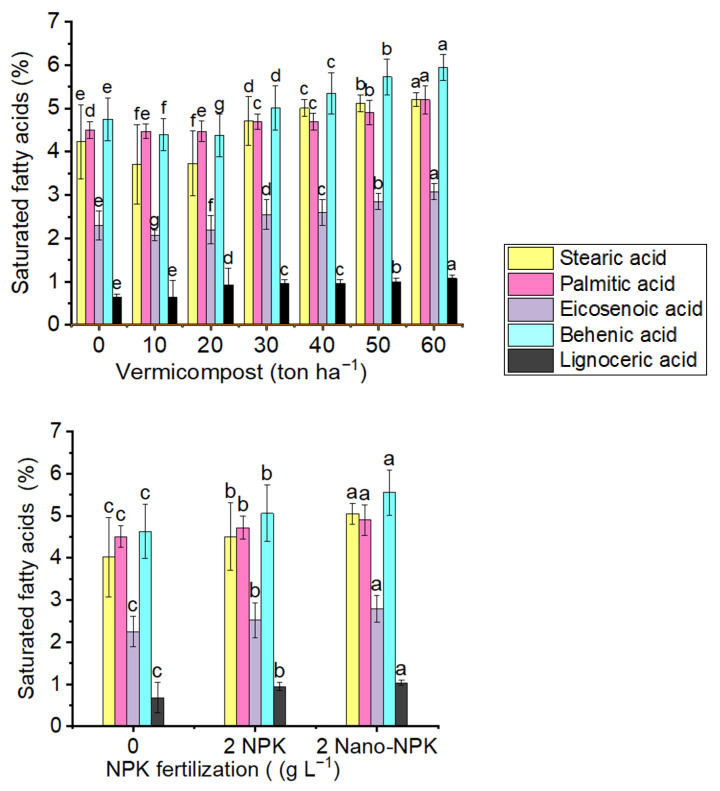
Means within the same saturated fatty acids (same color) with the same lowercase letters are not significantly different by the least significant difference (LSD) at *p* ≤ 0.05. Data are mean values ± SE (*n* = 3).

The major of detected saturated fatty acids of *Moringa oleifera* fixed oil are stearic acid, palmitic acid, eicosenoic acid, behenic acid and lignoceric acids (Figure 6 and Table 8). In this study, all saturated fatty acids of *Moringa oleifera* fixed oil increased by increasing the vermicompost level. The 10 ton ha^−1^ vermicompost treatment produced the minimum percentage of stearic acid (3.71%), palmitic acid (4.47%); eicosenoic acid (2.07%), behenic acid (4.40%), and lignoceric acid (0.64%). With NPK treatments, the control treatment had the minimum mean percentages of stearic acid (4.02%), palmitic acid (4.51%), eicosenoic acid (2.25%), behenic acid (4.63%) and lignoceric acid (0.68%). With the combination treatment, the minimum percentage of stearic acid (3.00%), palmitic acid (4.20%), eicosenoic acid (1.91%), behenic acid (3.81%) and lignoceric acid (0.14%) were recorded with the application of the 20 ton ha^−1^ vermicompost plus NPK control treatment.

#### 2.7.2. Unsaturated Fatty Acids

**Figure 7:**
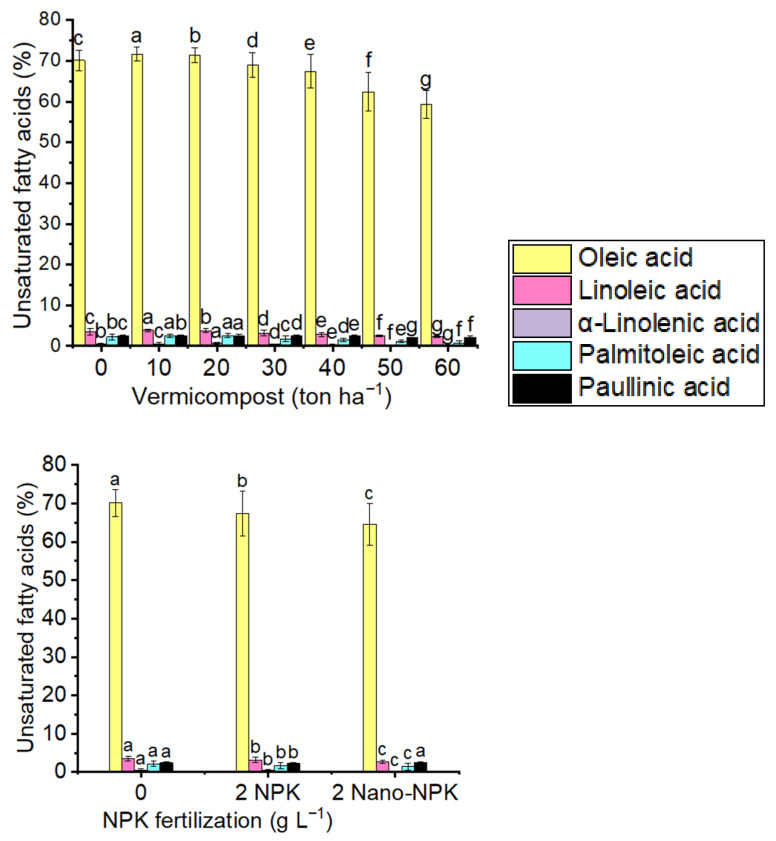
Means within the same unsaturated fatty acids (same color) with the same lowercase letters are not significantly different by the least significant difference (LSD) at *p* ≤ 0.05. Data are mean values ± SE (*n* = 3).

The main unsaturated fatty acids of *Moringa oleifera* fixed oil are oleic acid, linoleic acid, α-linolenic acid, palmitoleic acid and paullinic acids (Figure 7 and Table 9). By comparing the between different levels of vermicompost, the highest mean percentages of oleic acid (71.63%) and linoleic acid (3.89%) were recorded with the 10 ton ha^−1^ vermicompost treatment, while the maximum percentage of α-linolenic acid (0.65%), palmitoleic acid (2.55%), and paullinic acid (2.61%) were recorded with the 20 ton ha^−1^ vermicompost treatment. Using NPK, the control gave the maximum mean percentage of oleic acid (70.09%), linoleic acid (3.56%), α-linolenic acid (0.58%), palmitoleic acid (2.20%) and paullinic acid (2.45%). Regarding the combination treatments, the maximum highly significant mean percentages of oleic acid (72.46 and 72.56%) were found with the fourth and seventh combination treatments, respectively. In addition, the maximum mean percentages of linoleic acid (4.18%), α-linolenic acid (0.83%), palmitoleic acid (3.13%), and paullinic acid (2.78%) were recorded with the seventh combination treatment.

## 3. Discussion

### 3.1. Effects of Calcareous Soil

In this experiment, the seeds of the *Moringa oleifera* tree were able to germinate under the conditions of calcareous soil without any organic or inorganic fertilization treatment (control treatment, T1); however, after that the seedlings grew but very slowly. Finally, they produced small trees looking like thin branches with a few leaves with very small inflorescences. This gave the minimum mean values of pod and seed parameters, and yield of mature pods, seeds, and fixed oil per tree and per hectare in both seasons. This may have been due to the lack of accessibility of soil nutrients resources in the calcareous soil where nitrogen had been lost via leaching, deep percolation, or N transformations. Moreover, there was low availability of phosphorous and micronutrients have low availability and there was an imbalance among potassium, magnesium, calcium, and other elements [10,11,53], as well as inappropriate soil properties for plant growth [9]. These results were in agreement with Bavaresco and Poni [54], who found that the conditions of calcareous soil decreased P and K in different plant organs, which leads to a decrease in whole canopy photosynthesis, which would be reflected in the dry matter of plants and, finally, give low pods and seeds yields. Moreover, the decrease increased with increased levels of carbonate in the soil. Khan and Qasimwheat [55] reported that yields and most of the yield components of wheat crop, in pots and in the field experiment, also decreased because of the effect of calcareous soil. Semida et al. [56] found that untreated plants grown in saline calcareous soil had the lowest growth parameters, concentrations of total soluble sugars, free proline, as well as anthocyanin and photosynthetic efficiency. Aboukila et al. [12] found that the germination parameters of squash 15 days after sowing in calcareous soil, recorded the minimum values as compared with amendment with compost and spent grain. On the contrary, in this study, the control treatment was among the treatments that gave the maximum significant mean values of fixed oil percentage after the second treatment of 0 ton ha^−1^ vermicompost plus 2 gL^−1^ NPK, with insignificant differences between them, since the control treatment gave very small semi-atrophied seeds. As mentioned before, in calcareous soil, the amount of nitrogen and its availability are limited [10,11,54]. Under such conditions, the metabolism of grown plants changes more toward production of secondary metabolites with non-N-containing factors such as phenolics, fatty acids, and terpenoids [57], and decreased production of compounds with high N content such as proteins for growth according to the C/N balance hypothesis.

### 3.2. Effects of Vermicompost

It was obvious from the obtained results that the improvements of all the studied parameters of pods and seeds and the yield of pods, seeds, and fixed oil per tree and per hectare increased when the amount of vermicompost used was increased in the two seasons of the study. These improvements may have been due to the role of vermicompost in regulating growth by its natural auxins, gibberellins, and cytokinins contents, and increasing the availability of plant nutrients such as nitrogen, phosphorus, potassium and micro-elements in the soil through the mineralization of organic matter; increasing soluble forms of nutrients by improving the soil pH; as well as increasing the uptake of elements by roots [30,58,59]. In addition, using vermicompost improves physical and chemical properties of the calcareous soil. Vermicompost fertilizer is suitable for the long growth season of the *Moringa oleifera* tree, with its low rate of nutrients degradation speed [44,60]. The results of the present study agree with those of other studies that have shown that increased vermicompost consumption improved vegetative features, while it also improved dry matter content and flowering [47,48,61,62,63,64,65,66,67] along with fruiting, seeds, and oil yield. These findings agree with those of Arancon et al. [68], Arancon et al. [44], Atiyeh et al. [69], Liuc and Pank [70] and Muscolo et al. [71] who found that using vermicompost improved growth and flowering parameters as well as quantitative and qualitative parameters of strawberry, petunia, marigold and roman chamomile, and wild carrot. 

Even though increased vermicompost improved the yield of fixed oil as a result of improving plant growth, it decreased the percentage of fixed oil of seeds, which may be because under high N conditions, the metabolism of grown plants shifts towards production of N-compounds, with reduced production of secondary non-N-containing metabolites such as phenolics, fatty acids, and terpenoids [72,73]. Whereas Law-Ogbomo [74] found that applying poultry manure to Okra plants increased growth, fruit yield, as well as P, K, Na, and Mn contents, Ngo and Rumple [75] and Aryal and Tamrakar [76] reported that in most cases vermicompost was more favorable than manure and plant compost, as the application of vermicompost resulted in increased growth and yield as compared with farmyard manure.

### 3.3. Effects of NPK Fertilizer

An inadequate supply of nitrogen, phosphorus and potassium during crop growth is known to have a negative impact on the reproductive capability, growth, and yield of plants [77,78,79]. These elements are responsible for many enzymatic and metabolic activities as well as effective growth of seeds, pods, inflorescences shoots and roots. N has been shown to increase the number and size of fruits and overall yield [80]. Phosphorus is the main element in ATP, which is the energy unit of cells and it gives phosphorus bonds in DNA and RNA. P has an important role in improving rooting, flowering and seed development [81,82] as well as fruit set. K plays a main role in the CO_2_ assimilation rate in plants through its role in opening and closing stomata. Thus, it enhances photosynthesis and controls in the amount of glucose produced in plants and its translocation to seeds by controlling the enzymes of carbohydrate metabolism [83,84,85]. The results of this study show that, foliar application of nano-NPK surpassed ground application of NPK in achieving the best mean values of the studied growth characteristics, yield and chemical compounds of the *Moringa oleifera* tree as compared with the other ground application treatments and the control in both seasons under alkaline calcareous soil conditions. On the one hand, foliar fertilization has better potential to correct nutritional deficiencies in plants caused by the improper supply of nutrients to roots, and this practice is usually more economical and effective under alkaline calcareous soil conditions [86,87,88]. On the other hand, normal fertilizers are lost to the environment and cannot be absorbed by plants, causing not only substantial economic and resource losses, as well as very serious environmental pollution [89]. Nanofertilizers have shown promising results in optimum concentrations, as their size is in the nano-scale at a range of 1-100 nm, which allows them to penetrate into plant leaves, the basic units for photosynthesis, gas exchange and transpiration [90,91], and therefore they can reduces the needed amount of nutrients needed while increasing plant productivity [92]. Spraying nano-fertilizers can obviate the interaction of nutrients with water, microorganisms, and calcareous soil, and increase plant parameters and yield [18,93,94]. These results are in accordance with those of many researchers. Silberstein and Wittwer [95] and Dixon [96] suggested that foliar application improved nutrient efficiency and was the most effective way for growers to supply nutrients. Fuglier [7] found that the application of nitrogen and phosphorus to *Moringa* trees encouraged root development and leaf canopy growth. Liu and Lal [97] reported that synthesized nano-fertilizer improved biomass and production of *Glycine max.* Fagbenro [98], Ainika and Amans [99], Ghafariyan [100], Mahmoodzadeh [101], and Delfani [102] reported that crop growth, chemical composition, and yield parameters were found to respond significantly to compound NPK fertilizer application. Abdel-Aziz [91] reported that direct exposure of wheat plants to a specific type of nano-particles caused significant increases in all growth parameters and yield determined with optimum concentrations of nanosolution. Elshamy et al. [92], Farnia and Ghorbani [103], Oyedeji [104], Bărăscu [105], and Mokrani [106] compared growth, biomass, grain yield, photosynthetic pigments, chemical constituents, protein content, and fruits and lipid yield of plants with foliar application of nanofertilizers and normal NPK fertilizer, and they reported that all those were better with nanofertilizers application. Khalid and Shedeed [107] recorded that the highest values of vegetative growth characteristics of plant height, leaf number, branch number, capsule number, herb dry weight, and seed yield, and the highest values of chemical contents including fixed oil, total carbohydrate, soluble sugars, protein, potassium, and phosphorus contents with foliar application of NPK as compared with a control treatment and ground applications. Hasaneen and Abdel-aziz [108] found that the growth parameters of French bean plants increased with foliar application of either NPK nanoparticles or nano-engineered CNTs-NPK. Mokrani et al. [106] reported that the importance of NPK fertilizers was their role of supplying the necessary nutrients for plant growth. Soylu et al. [109], Soleimani [110], Arif et al. [111], and Hamayun et al. [112] reported rapid vegetative growth, and significant increases in the number of leaves, plant height, thousand-grain weight, and wheat yield as a result of foliar application of nitrogen, phosphorus, and potassium, together or individually. Jubeir and Ahmed [113] found that using nanofertilizer improved fruit weight, yield percentage at maturity, the appearance of amino acids in fruits, dry matter in leaves, and chlorophyll content, The treatment improved the vegetative growth and increased the yield of date palm. Alzreejawi and Al-Juthery [114] recorded the significant superiority of Nano-NPK (12-12-36) spray in achieving the highest means values for chlorophyll content in leaves, plant height, stem diameter, biological yield, grain yield, and harvest index. Rafiullah et al. [115] reported that fixation of phosphate fertilizers in alkaline calcareous soil was a major obstacle that could decrease the yield of maize and wheat. Foliar P on maize significantly enhanced grain yield and phosphorus use efficiency. 

### 3.4. Effects of Combination Treatments of Vermicompost and NPK

Despite the important role of foliar NPK application in terms of rapid assimilation and translocation and the positive influence on growth and yield, foliar fertilization cannot replace nutrition through the roots;. however, it can be used to reduce the use of fertilizers on the soil [116]. To overcome this problem, the use of organic amendments such as adding vermicompost plus applying foliar nano-NPK in the calcareous soil conditions is a good practices that can improve the growth; pod, seed, and fixed oil yield; and fatty acid content of the *Moringa oleifera* trees. 

In this study, combination treatments of vermicompost plus NPK and nano-NPK improved pod and seed parameters and yield per tree and per hectare, and these improvements increased with an increased vermicompost level and with spraying nano-NPK, while treatment of vermicompost control plus 2 gL^−1^ NPK gave the maximum fixed oil percentage. In addition, the minimum mean percentages of stearic acid, palmitic acid, eicosenoic acid, behenic acid and lignoceric saturated fatty acids and the maximum mean percentages of oleic acid, linoleic acid, α-linolenic acid, palmitoleic acid, and paullinic unsaturated fatty acids were recorded with the application of the combination treatment of 20 ton ha^−1^ vermicompost plus the NPK control. The combination of organic and inorganic fertilizers, generally, has vital effects on plant growth as well as soil chemical and biological properties [117,118] generally, and in this study foliar application of Nano-NPK combined with ground application of vermicompost mean availability of NPK and other required nutrients for seed quality and production. Therefore, using vermicompost alone or using vermicompost integrated with mineral fertilizers promoted plant growth and yield [119].

Our results were in agreement with those of Bajracharya et al. [120], Bhattarai and Tomar [121], Thakur [122], Zhao et al. [123], and Prativa and Bhattarai [124], who reported that the use of vermicompost in combination with NPK gave the best results in terms of plant growth and fruit yield. Despite the decreased fixed oil percentage of seeds with an increased fertilization level, the yield increased due to the ability of fertilization to increase seed yield. This was in agreement with Valiki et al. [119], Morshedi [125], Rogério et al. [126] and Xie et al. [127], who studied fennel, canola, crambe, and flax, respectively. Anwar et al. [128] noted that fixed oil of *Moringa oleifera* seeds was up to 40% with a high-quality fatty acid composition, as the percentage of unsaturated oleic fatty acid reached 70% or more. In this study, the percentage of oleic acid decreased with increased fertilization level. These results were in agreement with those of Xie et al. [129] on flax and Darakeh et al. [130] on black cumin.

## 4. Materials and Methods

The present investigation was carried out during two successive seasons in 2018/2019 and 2019/2020 in an open field of a private farm in El-Amiriya, Alexandria Governorate, Egypt. The aim wasto study the effects of organic and inorganic fertilization on pod, seed and fixed oil yield as well as composition, especially the percentage of oleic fatty acid of the *Moringa oleifera* trees. 

### 4.1. Plant Material

Seeds were collected from one selected mature *Moringa oleifera* tree grown alone in an isolated place in the study location. Its seed was previously brought from the national research center. The seeds were cultivated in February 2018 and 2019. A drip irrigation system was applied. Soil drainage conditions at the site were adequate to guarantee good oxygenation of the crop.

### 4.2. Treatment

The experiments were conducted in a split plot arranged in a Randomized Complete Block Design (RCBD) with three replications during February and March 2018/2019 and 2019/2020. The main plots of the *Moringa oleifera* plants were assigned to organic fertilization in the form of a ground dose of vermicompost and sub-plots were assigned to mineral fertilization in the forms of mineral and nano-NPK (19:19:19). All possible combinations of the two studied factors were made (Table 10). The experiments included 21 treatments, which were combinations of vermicompost added to the ground (0 (control), 10, 20, 30, 40, 50 or 60 ton ha^−1^ vermicompost, applied to the ground) and NPK fertilization (0 (control) and 2 gL^−1^ NPK or 2 gL^−1^ Nano-NPK). Every studied amount of vermicompost was added before planting over ten days, while the 2 gL^−1^ NPK treatment was applied as a ground dose and 2 gL^−1^ Nano-NPK was applied as foliar application. All NPK treatments were applied once per week after two weeks and until six weeks from planting; after that, they were applied twice a week until the end of the experiment. Tween 80 (0.01%) was used as the wetting agent. Untreated plants (NPK control) and plants treated with 2 gL^−1^ NPK were sprayed with distilled water and Tween 80 (0.01%). The data are presented as mean values ± SE (*n* = 3). 

### 4.3. Nano-NPK Preparation

Around 400 g of 19:19:19 NPK mineral fertilizer was weighed in a 2 L glass beaker, then 550 mL of distilled water was added, and it was stirred until completely dissolved. The clear solution was heated to 50 °C, and with vigorous stirring, 50 g of citric acid was added and stirring was continued for 15 min. Potassium hydroxide was added slowly until the desired pH was reached. During the addition of potassium hydroxide, the clear solution changed to a milky appearance, indicating the conversion to nanoparticle size. The concentration used was prepared according to the amount of mineral NPK used in the preparation of nano-NPK. Seeds were sown on 1^st^ February in both seasons. 

### 4.4. Culture of Seeds

Seeds (three seeds per hill^−1^) were sown on one side of the row. After 30 days, the seedlings were thinned to one plant per hill. The plots were weeded every two weeks when possible. The climate of the culture location is desert, with a mean annual temperature of 20.8 °C and annual precipitation of 181 mm, mainly falling in November through February [131]. A composite soil sample was collected at a depth of 0–30 cm from 15 different sites in the study, air-dried, and sieved through a 2-mm sieve prior to analysis. Sub-samples of the air-dried soil were used for chemical and physical parameters determination (Three sub-samples for every parameter). The physical and chemical properties of the vermicompost and soil samples were determined according to [132,133] as shown in Table 11 and Table 12 for every soil parameter (*n* = 3).

### 4.5. Parameters and Measurements

#### 4.5.1. Pods and Seeds Parameters and Yield

A sample of five plants was taken at random from each replication and fifteen plants from every treatment to measure the following parameters: number of pods per inflorescence, number of pods per tree, weight of mature pods (g), yield of mature pods (g tree^−1^ and kg ha^−1^), number of seeds per pod, weight of mature seed weight, and yield of mature seeds (g tree^−1^ and kg ha^−1^). The data are presented as mean values ± SE (*n* = 3). 

#### 4.5.2. Chemical Constituents of Seeds

##### Fixed Oil Content of Seeds

To determine the fixed oil content, seeds of each treatment were randomly selected, weighed, and dried at 50 °C. The drying process was continued until the difference between the two successive weights was less than 1 mg. Three replications were used for this process. The oil was extracted over 16 h with hexane using a Soxhlet apparatus [134]. The percentage of fixed oil was estimated, then, the fixed oil contents per plant and per hectare were calculated. The data are presented as mean values ± SE (*n* = 3). 

##### Fixed Oil Analysis (GC/MS Analysis)

Fatty acid methyl esters were prepared with methanolic sulfuric acid and characterized by gas chromatography mass spectrometry. The analyses of the fixed oil were conducted using a gas chromatography–mass spectrometry (GC-MS) instrument at the Department of Medicinal and Aromatic Plants Research, National Research Center as mentioned in Atteya and Amer [135]

### 4.6. Statistical Analysis

The experiments were a split plot arranged in a Randomized Complete Block Design (RCBD) with three replicates. Analysis of variance with SAS software [136] was carried out on all tested treatments data. means of treatments were compared using the LSD test at 5% level of probability. The experiment was repeated in the second year using the same steps and techniques of the first year to compare the results in the two successive seasons. 

## 5. Conclusions

As Compared with the soil-application of NPK, in this study, the foliar application of nano-NPK provided a good resolution for a low availability of NPK. Moreover, increasing the amount of vermicompost improved the parameters and yield of *Moringa oleifera* pods and seeds. Finally, in this study, the recommended treatment for reaching the maximum values for the yield of mature pods, seeds, and fixed oil per tree and per hectare is the 50 ton ha^−1^ vermicompost plus 2 g L^−1^ Nano-NPK treatment. The 20 ton ha^−1^ vermicompost plus NPK control treatment is recommended for producing fixed oil with the minimum percentage of saturated fatty acids and the maximum percentage of oleic acid.

## Figures and Tables

**Table 1:** The mean values of the number of pods per inflorescence of the *Moringa oleifera* tree as affected by combination treatments of organic and mineral fertilization, in both seasons of the study.

Treatments	Number of Pods per Inflorescence	
	1st Season	2nd Season
T1	0.88 ± 0.06 r	0.92 ± 0.01 u
T2	0.98 ± 0.07 r	1.02 ± 0.01 t
T3	1.77 ± 0.13 n	1.84 ± 0.02 p
T4	1.18 ± 0.08 q	1.23 ± 0.02 s
T5	1.37 ± 0.10 p	1.43 ± 0.02 r
T6	2.26 ± 0.16 kl	2.36 ± 0.03 m
T7	1.57 ± 0.11 o	1.64 ± 0.02 q
T8	2.36 ± 0.17 k	2.46 ± 0.03 l
T9	2.75 ± 0.20 hi	2.87 ± 0.04 i
T10	1.96 ± 0.14 m	2.05 ± 0.03 o
T11	2.55 ± 0.18 j	2.66 ± 0.03 k
T12	3.04 ± 0.22 ef	3.18 ± 0.04 f
T13	2.16 ± 0.16 l	2.25 ± 0.03 n
T14	2.95 ± 0.21 fg	3.07 ± 0.04 g
T15	3.34 ± 0.24 bc	3.48 ± 0.04 c
T16	2.65 ± 0.19 ij	2.77 ± 0.03 j
T17	3.14 ± 0.23 de	3.28 ± 0.04 e
T18	3.44 ± 0.25 ab	3.59 ± 0.05 b
T19	2.85 ± 0.21 gh	2.97 ± 0.04 h
T20	3.24 ± 0.23 cd	3.38 ± 0.04 d
T21	3.53 ± 0.25 a	3.69 ± 0.05 a

Means in columns followed by the same lowercase letters are not statistically different at the 0.05 significance level. Data are mean values ± SE (*n* = 3).

**Table 2:** The mean values of the number of pods per tree and the weight of mature pods (g) of the *Moringa oleifera* tree as affected by combination treatments of organic and mineral fertilization, in both seasons of the study.

Treatments	Number of Pods per Tree		Weight of Mature Pods (g)	
	1st Season	2nd Season	1st Season	2nd Season
T1	5.6 ± 0.9 l	6.0 ± 0.1 s	4.39 ± 0.09 u	4.27 ± 0.35 m
T2	9.2 ± 1.5 kl	9.8 ± 0.2 rs	5.30 ± 0.10 t	5.17 ± 0.42 l
T3	29.3 ± 5.0 ijk	31.4 ± 0.8 o	6.43 ± 0.13 p	6.26 ± 0.51 k
T4	11.6 ± 1.9 kl	12.4 ± 0.3 r	5.51 ± 0.11 s	5.36 ± 0.44 l
T5	19.0 ± 3.2 jkl	20.4 ± 0.5 q	5.71 ± 0.11 r	5.56 ± 0.46 l
T6	54.5 ± 9.3 gh	58.4 ± 1.4 l	7.65 ± 0.15 m	7.45 ± 0.61 i
T7	23.7 ± 4.0 ijkl	25.4 ± 0.6 p	6.22 ± 0.12 q	6.06 ± 0.50 k
T8	63.3 ± 10.9 fg	67.9 ± 1.7 k	8.06 ± 0.16 l	7.85 ± 0.64 hi
T9	95.2 ± 16.4 e	102.0 ± 2.5 h	8.98 ± 0.18 i	8.74 ± 0.72 efj
T10	35.8 ± 6.1 hij	38.4 ± 0.9 n	6.94 ± 0.14 o	6.75 ± 0.55 j
T11	76.6 ± 13.2 ef	82.1 ± 2.0 j	8.36 ± 0.16 k	8.15 ± 0.67 gh
T12	132.8 ± 23.0 d	142.4 ± 3.5 e	9.69 ± 0.19 f	9.44 ± 0.77 d
T13	42.6 ± 7.2 ghi	45.6 ± 1.1 m	7.14 ± 0.14 n	6.95 ± 0.57 j
T14	117.3 ± 20.3 d	125.8 ± 3.1 f	9.49 ± 0.19 g	9.24 ± 0.76 d
T15	207.0 ± 36.0 b	209.1 ± 5.2 c	10.20 ± 0.20 c	9.93 ± 0.81 c
T16	85.3 ± 14.7 e	94.1 ± 3.9 i	8.77± 0.17 j	8.54 ± 0.70 fg
T17	164.7 ± 28.6 c	170.7 ± 4.2 d	9.79 ± 0.19 e	9.92 ± 0.15 c
T18	243.8 ± 41.1 a	263.5 ± 6.6 a	10.71± 0.21 b	10.43 ± 0.86 b
T19	95.4 ± 9.4 e	116.3 ± 4.4 g	9.28 ± 0.18 h	9.04 ± 0.74 de
T20	158.0 ± 27.4 c	167.2 ± 4.1 d	10.00 ± 0.20 d	9.31 ± 0.69 d
T21	202.6 ± 38.6 b	226.6 ± 8.1 b	11.22 ± 0.22 a	10.93 ± 0.90 a

Means in columns followed by the same lowercase letters are not statistically different at the 0.05 significance level. Data are mean values ± SE (*n* = 3).

**Table 3:** The mean value of yield of mature pods (g tree^−1^) and yield of mature pods (kg ha^−1^) of the *Moringa oleifera* tree as affected by combination treatments of organic and mineral fertilization in both seasons of the study.

Treatments	Yield of Mature Pods (g Tree^−1^)		Yield of Mature Pods (kg ha^−1^)	
	1st Season	2nd Season	1st Season	2nd Season
T1	25 ± 4 l	26 ± 2 o	246 ± 43 l	256 ± 24 o
T2	49 ± 9 l	51 ± 5 no	489 ± 88 kl	509 ± 48 no
T3	189 ± 35 jkl	197 ± 19 lmn	1888 ± 349 jkl	1966 ±186 lmn
T4	64 ±12 kl	66 ± 6 no	639 ± 116 kl	665 ± 63 no
T5	109 ± 20 kl	113 ± 11mno	1088 ± 200 kl	1133 ± 107 mno
T6	418 ± 78 hij	436 ± 41 jk	4180 ± 781 hij	4356 ± 412 jk
T7	148 ± 27 kl	154 ± 15 mno	1478 ± 272 jkl	1539 ± 145 mno
T8	511 ± 96 ghi	533 ± 50 ij	5112 ± 958 ghi	5328 ± 503 ij
T9	856 ± 161 f	892 ± 84 g	8560 ± 1611 ef	8922 ± 843 g
T10	249 ± 46 jkl	260 ± 25 lm	2491 ± 462 ijkl	2595 ± 245 lm
T11	642 ± 121 fgh	669 ± 63 hi	6421 ± 1206 fgh	6692 ± 632 hi
T12	1289 ± 243 d	1344 ± 127 e	12894 ± 2434 d	13441 ± 1271 e
T13	305 ± 57 ijk	317 ± 30 kl	3046 ± 566 ijk	3173 ± 300 kl
T14	1115 ± 210 de	1162 ± 110 f	11151 ± 2103 de	11624 ± 1099 f
T15	2116 ± 401 b	2078 ± 197 c	21157 ± 4009 b	20783 ± 1965 c
T16	750 ± 141 fg	806 ± 97 gh	7498 ± 1409 fg	8058 ± 970 gh
T17	1616 ± 306 c	1692 ± 46 d	16160 ± 3057 c	16924 ± 462 d
T18	2616 ± 482 a	2750 ± 260 a	26158 ± 4820 a	27496 ± 2600 a
T19	887 ± 103 ef	1054 ± 123 f	8867 ± 1031 ef	10536 ± 1231 f
T20	1583 ± 299 c	1559 ± 155 d	15826 ± 2992 c	15586 ± 1546 d
T21	2276 ± 473 b	2479 ± 271b	22758 ± 4727 a	24795 ± 2708 b

Means in columns followed by the same lowercase letters are not statistically different at the 0.05 significance level. Data are mean values ± SE (*n* = 3).

**Table 4:** The mean values of the number of seeds per pod and weight of mature seed weight (g) of the *Moringa oleifera* tree as affected by combination treatments of organic and mineral fertilization in both seasons of the study.

Treatments	Number of Seeds per Pod		Mature Seed Weight(g)	
	1st Season	2nd Season	1st Season	2nd Season
T1	5.1 ± 0.1 u	5.2 ± 0.1 u	0.031 ± 0.001 u	0.031 ± 0.001 u
T2	9.2 ± 0.2 t	9.3 ± 0.2 t	0.082 ± 0.002 t	0.083 ± 0.002 t
T3	15.7 ± 0.3 p	15.9 ± 0.3 p	0.123 ± 0.003 p	0.125 ± 0.002 p
T4	10.2 ± 0.2 s	10.3 ± 0.2 s	0.093 ± 0.002 s	0.094 ± 0.002 s
T5	13.3 ± 0.2 r	13.4 ± 0.3 r	0.103 ± 0.002 r	0.104 ± 0.002 r
T6	17.2 ± 0.3 m	17.3 ± 0.3 m	0.155 ± 0.003 m	0.157 ± 0.003 m
T7	15.3 ± 0.3 q	15.5 ± 0.3 q	0.113 ± 0.002 q	0.114 ± 0.002 q
T8	17.4 ± 0.3 l	17.5 ± 0.3 l	0.162 ± 0.004 l	0.164 ± 0.003 l
T9	18.4 ± 0.3 i	18.5 ± 0.4 i	0.185 ± 0.004 i	0.187 ± 0.004 i
T10	16.3 ± 0.3 o	16.5 ± 0.3 o	0.144 ± 0.003 o	0.146 ± 0.003 o
T11	17.6 ± 0.3 k	17.7 ± 0.3 k	0.166 ± 0.004 k	0.167 ± 0.003 k
T12	19.4 ± 0.3 f	19.6 ± 0.4 f	0.221 ± 0.005 f	0.224 ± 0.004 f
T13	16.7 ± 0.3 n	16.8 ± 0.3 n	0.151 ± 0.003 n	0.153 ± 0.003 n
T14	19.0 ± 0.3 g	19.2 ± 0.4 g	0.195 ± 0.004 g	0.198 ± 0.004 g
T15	20.3 ± 0.3 c	20.5 ± 0.4 c	0.247 ± 0.005 c	0.250 ± 0.005 c
T16	18.1 ± 0.3 j	18.2 ± 0.4 i	0.180 ± 0.004 i	0.182 ± 0.004 i
T17	19.7 ± 0.3 e	19.9 ± 0.4 e	0.216 ± 0.005 e	0.218 ± 0.004 e
T18	21.5 ± 0.4 b	21.6 ± 0.4 b	0.257 ± 0.006 b	0.260 ± 0.005 b
T19	18.7 ± 0.3 h	18.8 ± 0.4 h	0.190 ± 0.004 h	0.192 ± 0.004 h
T20	20.0 ± 0.3 d	20.2 ± 0.4 d	0.226 ± 0.005 d	0.229 ± 0.004 d
T21	22.8 ± 0.4 a	23.0 ± 0.4 a	0.267 ± 0.006 a	0.270 ± 0.005 a

Means in columns followed by the same lowercase letters are not statistically different at the 0.05 significance level. Data are mean values ± SE (*n* = 3).

**Table 5:** The mean values of mature seeds yield (g tree^−1^) and mature seeds yield (kg ha^−1^) of the *Moringa oleifera* tree as affected by combination treatments of organic and mineral fertilization in both seasons of the study.

Treatments	Yield of Mature Seeds (g Tree^−1^)		Yield of Mature Seeds (kg ha^−1^)	
	1st Season	2nd Season	1st Season	2nd Season
T1	1 ± 0 l	1 ± 0 p	9 ± 2 l	10 ± 0 p
T2	7 ± 1 l	8 ± 0 op	70 ± 13 l	76 ± 4 op
T3	57 ± 11 jkl	62 ± 3 mn	572 ± 113 jkl	622 ± 30 mn
T4	11 ± 2 kl	12 ± 1 op	110 ± 21 kl	119 ± 6 op
T5	26 ± 5 kl	28 ± 1 nop	261 ± 51 kl	284 ± 14 nop
T6	146 ± 29 hijk	159 ± 8 jk	1460 ± 290 hijk	1588 ± 77 jk
T7	41 ± 8 kl	45 ± 2 no	413 ± 81 kl	449 ± 22 no
T8	179 ± 36 hij	195 ± 9 j	1794 ± 358 hijk	1953 ± 95 j
T9	325 ± 65 fg	354 ± 17 h	3253 ± 651 fg	3541 ± 172 h
T10	85 ± 17 jkl	92 ± 4 lm	847 ± 167 jkl	922 ± 45 lm
T11	224 ± 45 ghi	244 ± 12 i	2238 ± 447 ghi	2436 ± 118 i
T12	572 ± 115 de	623 ± 30 e	5723 ± 1149 de	6231 ± 302 e
T13	108 ± 21 ijkl	117 ± 6 kl	1076 ± 213 ijkl	1171 ± 57 kl
T14	437 ± 88 ef	476 ± 23 f	4374 ± 877 ef	4763 ± 231 f
T15	1043 ± 210 b	1070 ± 55 c	10431 ± 2100 b	10701 ± 551 c
T16	279 ± 56 gh	313 ± 15 h	2788 ± 557 gh	3127 ± 147 h
T17	704 ± 127 cd	741 ± 33 d	7043 ± 1273 cd	7413 ± 335 d
T18	1350 ± 265 a	1482 ± 70 a	13501 ± 2654 a	14821 ± 696 a
T19	340 ± 76 fg	423 ± 20 g	3403 ± 757 fg	4226 ± 200 g
T20	719 ± 160 c	772 ± 42 d	7189 ± 1597 c	7723 ± 419 d
T21	1237 ± 323 a	1408 ± 85 b	12372 ± 3232 a	14083 ±8 47 b

Means in columns followed by the same lowercase letters are not statistically different at the 0.05 significance level. Data are mean values ± SE (*n* = 3).

**Table 6:** The mean values of fixed oil percentage (%) and fixed oil content (mL plant^−1^) of *Moringa oleifera* seeds as affected by combination treatments of organic and mineral fertilization in both seasons of the study.

Treatments	Fixed Oil Percentage (%)		Fixed Oil Content (mL Plant^−1^)	
	1st Season	2nd Season	1st Season	2nd Season
T1	36.57 ± 3.07 a	36.49 ± 2.40 ab	0.3 ± 0.1 h	0.4 ± 0.0 r
T2	38.69 ± 1.22 a	37.48 ± 2.12 a	4.2 ± 0.7 gh	4.5 ± 0.3 r
T3	32.41 ± 6.29 b	35.52 ± 1.84 bc	17.8 ± 0.9 fgh	22.6 ± 1.1 o
T4	37.49 ± 2.82 a	36.78 ± 2.09 ab	2.6 ± 0.4 h	2.8 ± 0.1 r
T5	36.55 ± 0.71 a	37.83 ± 1.50 a	9.5 ± 2.0 gh	10.7 ± 0.2 q
T6	29.17± 0.91 cd	33.78 ± 1.10 c	42.5 ± 8.1 ef	53.6 ± 1.1 l
T7	37.22 ± 1.16 a	36.54 ± 1.19 ab	15.3 ± 2.9 fgh	16.4 ± 0.3 p
T8	36.72 ± 1.15 a	36.05 ± 1.18 ab	65.7 ± 12.5 de	70.3 ± 1.4 k
T9	28.17 ± 0.88 cd	28.64 ± 0.93 ef	91.4 ± 17.5 cd	101.3 ± 2.0 h
T10	36.89 ± 1.71 a	35.14 ± 1.14 bc	32.2 ± 6.1 fg	32.4 ± 1.0 n
T11	32.19 ± 1.01 b	31.61 ± 1.03 d	71.9 ± 13.7 d	76.9 ± 1.5 J
T12	26.98 ± 0.40 de	27.65 ± 0.90 fg	154.4 ± 31.1 b	172.2 ± 3.4 e
T13	36.22 ± 1.13 a	35.56 ± 1.16 bc	38.9 ± 7.4 ef	41.6 ± 0.8 m
T14	23.14 ± 0.72 fg	22.72 ± 0.74 ij	101.0 ± 19.3 c	108.1 ± 2.2 g
T15	25.15 ± 0.79 ef	24.69 ± 0.80 h	261.8 ± 50.4 a	264.0 ± 5.3 c
T16	30.18 ± 0.94 bc	29.63 ± 0.97 e	83.9 ± 16.0 cd	92.5 ± 4.4 i
T17	24.79 ± 0.69 ef	24.07 ± 1.24 hi	174.8 ± 37.4 b	178.2 ± 2.0 d
T18	20.06 ± 0.31 h	20.21 ± 0.89 k	270.5 ± 52.2 a	299.1 ± 2.3 a
T19	27.16 ± 0.85 de	26.67 ± 0.87 g	92.3 ± 10.8 cd	112.5 ± 5.0 g
T20	21.44 ± 0.95 gh	21.11 ± 0.73 jk	153.3 ± 26.1 b	163.0 ± 7.9 f
T21	20.01 ± 0.17 h	20.09 ± 0.84 k	247.4 ± 27.8 a	282.4 ± 7.2 b

Means in columns followed by the same lowercase letters are not statistically different at the 0.05 significance level. Data are mean values ± SE (*n* = 3).

**Table 7:** The mean values of yield of fixed oil (L ha^−1^) of *Moringa oleifera* seeds as affected by combination treatments of organic and mineral fertilization in both seasons of the study.

Treatments	Yield of Fixed Oil (L ha^−1^)	
	1st Season	2nd Season
T1	3 ± 1 h	4 ± 0 r
T2	42 ± 7 gh	45 ± 3 r
T3	178 ± 9 fgh	226 ± 11 o
T4	26 ± 4 h	28 ± 1 r
T5	95 ± 20 gh	107 ± 2 q
T6	425 ± 81 ef	536 ± 11 l
T7	153 ± 29 fgh	164 ± 3 p
T8	657 ± 125 de	703 ± 14 k
T9	914 ± 175 cd	1013 ± 20 h
T10	322 ± 61 fg	324 ± 10 n
T11	719 ± 137 d	769 ± 15 j
T12	1544 ± 311 b	1722 ± 34 e
T13	389 ± 74 ef	416 ± 8 m
T14	1010 ± 193 c	1081 ± 22 g
T15	2618 ± 504 a	2640 ± 53 c
T16	839 ± 160 cd	925 ± 44 i
T17	1748 ± 374 b	1782 ± 20 d
T18	2705 ± 522 a	2991 ± 23 a
T19	923 ± 108 cd	1125 ± 50 g
T20	1533 ± 261 b	1630 ± 79 f
T21	2474 ± 278 a	2824 ± 72 b

Means in columns followed by the same lowercase letters are not statistically different at the 0.05 significance level. Data are mean values ± SE (*n* = 3).

**Table 8:** The mean percent of saturated fatty acids (%) of fixed oil of *Moringa oleifera* seeds as affected by combination treatments of organic and mineral fertilization.

Treatments	Stearic Acid	Palmitic Acid	Eicosenoic Acid	Behenic Acid	Lignoceric Acid
T1	3.12 ± 0.10 i	4.31 ± 0.15 j	2.01 ± 0.06 n	4.26 ± 0.14 n	0.84 ± 0.02 g
T2	4.61 ± 0.16 f	4.56 ± 0.15 h	2.16 ± 0.07 j	4.64 ± 0.16 j	0.92 ± 0.02 def
T3	4.96 ± 0.17 c	4.64 ± 0.16 f	2.72 ± 0.09 e	5.35 ± 0.18 e	1.00 ± 0.03 c
T4	3.03 ± 0.10 j	4.32 ± 0.15 j	1.97 ± 0.06 o	4.02 ± 0.13 o	0.15 ± 0.10 h
T5	3.16 ± 0.10 i	4.51 ± 0.15 i	2.04 ± 0.06 m	4.36 ± 0.15 m	0.88 ± 0.02 fg
T6	4.9 ± 0.17 cd	4.57 ± 0.16 h	2.20 ± 0.07 i	4.83 ± 0.16 i	0.94± 0.02 de
T7	3.00 ± 0.10 j	4.20 ± 0.14 k	1.91 ± 0.06 p	3.81 ± 0.13 p	0.14 ± 0.10 h
T8	3.51 ± 0.12 h	4.59 ± 0.16 g	2.10 ± 0.07 l	4.41 ± 0.15 l	0.86 ± 0.02 g
T9	4.67 ± 0.16 e	4.63 ± 0.16 f	2.61 ± 0.08 g	4.9 ± 0.17 gh	0.9 ± 0.02 cd
T10	4.01 ± 0.13 g	4.60 ± 0.16 g	2.14 ± 0.07 k	4.53 ± 0.15 k	0.85 ± 0.02 g
T11	4.94± 0.17 cd	4.64 ± 0.16 f	2.63 ± 0.08 fg	4.87 ± 0.17 hi	1.00 ± 0.03 c
T12	5.18± 0.18 ab	4.85 ± 0.17 d	2.88 ± 0.09 d	5.63 ± 0.19 d	1.05 ± 0.03 b
T13	4.90 ± 0.17 d	4.59 ± 0.16 g	2.23 ± 0.07 h	4.92 ± 0.17 g	0.91 ± 0.02 ef
T14	4.95± 0.17 cd	4.64 ± 0.16 f	2.65 ± 0.09 f	5.20 ± 0.18 f	0.88 ± 0.02 fg
T15	5.19± 0.18 ab	4.88 ± 0.17 c	2.90 ± 0.09 d	5.94 ± 0.20 c	1.07± 0.03 ab
T16	4.9 ± 0.17 cd	4.66 ± 0.16 e	2.64 ± 0.08 f	5.23 ± 0.18 f	0.86 ± 0.02 g
T17	5.20± 0.18 ab	4.88 ± 0.17 c	2.95 ± 0.10 c	5.91 ± 0.20 c	1.05 ± 0.03 b
T18	5.20± 0.18 ab	5.20 ± 0.18 b	2.97 ± 0.10 c	6.04 ± 0.21 b	1.08± 0.03 ab
T19	5.17 ± 0.18 b	4.87 ±0.17 c	2.89 ± 0.09 d	5.62 ± 0.19 d	1.06 ± 0.03 b
T20	5.22± 0.18 ab	5.21 ± 0.18 b	3.13 ± 0.10 b	6.06 ± 0.21 b	1.08± 0.03 ab
T21	5.23 ± 0.18 a	5.52 ± 0.19 a	3.23 ± 0.11 a	6.15 ± 0.21 a	1.11 ± 0.03 a

Means in columns followed by the same letter are not statistically different at the 0.05 significance level. Data are mean values ± SE (*n* = 3).

**Table 9:** The mean percent of unsaturated fatty acids (%) of fixed oil of *Moringa oleifera* seeds as affected by combination treatments of organic and mineral fertilization.

Treatments	Oleic Acid	Linoleic Acid	α-Linolenic Acid	Palmitoleic Acid	Paullinic Acid
T1	71.87 ± 1.80 b	4.03 ± 0.09 c	0.72 ± 0.01 c	2.34 ± 0.05 f	2.43 ± 0.05 h
T2	70.97 ± 1.78 d	3.83 ± 0.09 g	0.56 ± 0.01 g	3.04 ± 0.07 b	2.27 ± 0.05 j
T3	67.48 ± 1.69 j	2.66 ± 0.06 n	0.31 ± 0.01 l	1.31 ± 0.02 k	2.76 ± 0.06 b
T4	72.46 ± 1.82 a	4.11 ± 0.09 b	0.79 ± 0.01 b	2.56 ± 0.05 d	2.72 ± 0.06 c
T5	71.77 ± 1.80 b	4.01 ± 0.09 d	0.66 ± 0.01 d	2.12 ± 0.04 g	2.49 ± 0.05 g
T6	70.67 ± 1.77 e	3.53 ± 0.08 h	0.05 ± 0.05 q	3.03 ± 0.07 b	2.38 ± 0.05 i
T7	72.56 ± 1.82 a	4.18 ± 0.09 a	0.83 ± 0.01 a	3.13 ± 0.07 a	2.78 ± 0.06 a
T8	71.47 ± 1.79 c	3.98 ± 0.09 e	0.64 ± 0.01 e	2.42 ± 0.05 e	2.38 ± 0.05 i
T9	70.27 ± 1.76 f	3.23 ± 0.07 j	0.48 ± 0.00 i	2.12 ± 0.04 g	2.68 ± 0.06 d
T10	71.37 ± 1.79 c	3.93 ± 0.09 f	0.61 ± 0.01 f	2.63 ± 0.06 c	2.18 ± 0.04 k
T11	69.97 ± 1.76 g	3.03 ± 0.07 k	0.41 ± 0.00 j	1.35 ± 0.02 j	2.58 ± 0.05 f
T12	65.49 ± 1.64 k	2.63 ± 0.06 o	0.29 ± 0.01 m	1.21 ± 0.02 l	2.64 ± 0.06 e
T13	70.47 ± 1.77ef	3.43 ± 0.08 i	0.50 ± 0.00 h	2.04 ± 0.04 h	2.49 ± 0.05 g
T14	69.47 ± 1.74 h	2.83 ± 0.06 l	0.34 ± 0.01 k	1.22 ± 0.02 l	2.09 ± 0.04 m
T15	62.40± 1.56 m	2.42 ± 0.05 q	0.02 ± 0.00 r	1.14 ± 0.02 m	2.68 ± 0.06 d
T16	68.48 ± 1.72 i	2.73 ± 0.06 m	0.32 ± 0.01 l	1.52 ± 0.03 i	2.07 ± 0.04 n
T17	60.40 ± 1.51 n	2.38 ± 0.05 r	0.20 ± 0.01 o	1.06 ± 0.02 n	2.16 ± 0.04 l
T18	58.41 ± 1.46 o	2.32 ± 0.05 s	0.19 ± 0.01 o	0.92 ± 0.01 o	2.06 ± 0.04 n
T19	63.39 ± 1.59 l	2.53 ± 0.05 p	0.27 ± 0.01 n	1.20 ± 0.02 l	2.49 ± 0.05 g
T20	57.41 ± 1.44 p	2.22 ± 0.05 t	0.14 ± 0.01 p	0.88 ± 0.01 p	2.01 ± 0.04 o
T21	57.02 ± 1.43 q	2.12 ± 0.04 u	0.02 ± 0.00 r	0.07 ± 0.05 q	1.98 ± 0.04 p

Means in columns followed by the same lowercase letters are not statistically different at the 0.05 significance level. Data are mean values ± SE (*n* = 3).

**Table 10:** All the different used combinations treatments of vermicompost and NPK fertilization with the following.

Treatments	
T1	Vermicompost control plus NPK control (Control)
T2	Vermicompost control plus 2 gL^−1^ NPK
T3	Vermicompost control plus 2 gL^−1^ Nano-NPK
T4	10 ton ha^−1^ vermicompost plus NPK control
T5	10 ton ha^−1^ vermicompost plus 2 gL^−1^ NPK
T6	10 ton ha^−1^ vermicompost plus 2 gL^−1^ Nano-NPK
T7	20 ton ha^−1^ vermicompost plus NPK control
T8	20 ton ha^−1^ vermicompost plus 2 gL^−1^ NPK
T9	20 ton ha^−1^ vermicompost plus 2 gL^−1^ Nano-NPK
T10	30 ton ha^−1^ vermicompost plus NPK control
T11	30 ton ha^−1^ vermicompost plus 2 gL^−1^ NPK
T12	30 ton ha^−1^ vermicompost plus 2 gL^−1^ Nano-NPK
T13	40 ton ha^−1^ vermicompost plus NPK control
T14	40 ton ha^−1^ vermicompost plus 2 gL^−1^ NPK
T15	40 ton ha^−1^ vermicompost plus 2 gL^−1^ Nano-NPK
T16	50 ton ha^−1^ vermicompost plus NPK control
T17	50 ton ha^−1^ vermicompost plus 2 gL^−1^ NPK
T18	50 ton ha^−1^ vermicompost plus 2 gL^−1^ Nano-NPK
T19	60 ton ha^−1^ vermicompost plus NPK control
T20	60 ton ha^−1^ vermicompost plus 2 gL^−1^ NPK
T21	60 ton ha^−1^ vermicompost plus 2 gL^−1^ Nano-NPK

**Table 11:** The physical and chemical properties of the used vermicompost.

Vermicompost Property		
Organic matter	%	44.57
C	%	17.02
N	%	1.82
Mn	%	0.03
B	mg g^−1^	0.054
Cu	mg g^−1^	0.25
Fe	mg g^−1^	1.27
Mg	mg g^−1^	6.01
Na	mg g^−1^	1.48
P_2_O_5_	mg g^−1^	4.61
K	mg g^−1^	1.93
EC	ds m^−1^	1.78
pH		7.2

**Table 12:** The physical and chemical properties of the experimental soil.

Soil Property		
Organic matter		0.75
CaCO_3_	%	28.62
Sand	%	65.3
Silt	%	15.8
Clay	%	18.9
Texture class	Sandy clay loam	
pH		8.51
EC	ds m^−1^	1.72
N	%	0.032
HCO_3_^-^	mg g^−1^	0.099
P_2_O_4_	mg g^−1^	0.004
K^+^	mg g^−1^	0.287
Fe	mg g^−1^	0.0038
Zn	mg g^−1^	0.0014
Mn	mg g^−1^	0.0035
Cu	mg g^−1^	0.00059
B	mg g^−1^	0.0003

## Data Availability

The data presented in this study are available within the article.
